# Green Synthesis of Ca-Doped ZnO Nanosheets with Tunable Band Structure via Cactus-Juice-Mediated Coprecipitation for Enhanced Photocatalytic H_2_ Evolution

**DOI:** 10.3390/molecules31071091

**Published:** 2026-03-26

**Authors:** Heji Luo, Huifang Liu, Simin Liu, Haiyan Wang, Lingling Liu, Xibao Li

**Affiliations:** 1 School of Materials Science and Engineering, Nanchang Hangkong University, Nanchang 330063, China; 18610228212@163.com (H.L.); 18839318760@163.com (H.L.); 13697048671@163.com (S.L.); 70833@nchu.edu.cn (H.W.); 2 College of Environment and Chemical Engineering, Nanchang Hangkong University, Nanchang 330063, China; 70628@nchu.edu.cn

**Keywords:** zinc oxide, calcium doping, cactus juice, coprecipitation, photocatalytic hydrogen production

## Abstract

The development of efficient, stable, and sustainably fabricated photocatalysts for solar-driven hydrogen evolution remains a critical challenge in the field. Herein, we report a novel green coprecipitation strategy to synthesize calcium-doped zinc oxide (Ca-ZnO) nanosheets, utilizing cactus juice as a natural, multifunctional medium for the coprecipitation process. This method enables the in situ, tunable incorporation of 3–7% Ca^2+^ ions into the wurtzite ZnO lattice without the use of harsh chemical reagents. Comprehensive characterization confirms that Ca^2+^ substitutionally replaces Zn^2+^, which preserves the intrinsic crystal structure of ZnO well while inducing the formation of uniform nanosheet morphology. This doping strategy effectively modulates the electronic band structure, progressively narrowing the bandgap from 3.19 eV to 2.90 eV and significantly enhancing visible-light absorption. Crucially, the incorporation of Ca^2+^ also generates oxygen vacancies, which serve as efficient electron traps to suppress photogenerated charge carrier recombination. The optimized 5%Ca-ZnO photocatalyst demonstrates a favorable hydrogen evolution rate of 889 μmol·g^−1^·h^−1^ under full-spectrum irradiation, with stability, retaining 94.8% of its activity after four cycles. This work not only provides a high-performance material but also establishes a generalizable, sustainable paradigm for the design of advanced semiconductor photocatalysts.

## 1. Introduction

Hydrogen is widely acknowledged as a promising energy carrier due to its clean combustion and high energy density, providing a feasible solution to mitigate the global energy crisis and environmental degradation. Photocatalytic water splitting is an effective strategy to convert solar energy into chemical fuels, in which the rational design of semiconductor photocatalysts represents the core of this technology [1,2,3]. Among various semiconductor materials, zinc oxide (ZnO) has garnered extensive research interest owing to its improved chemical stability, excellent charge carrier mobility, good environmental compatibility, and low preparation cost. Nevertheless, its practical application is hindered by a relatively large bandgap (~3.37 eV), which leads to limited visible-light harvesting, as well as rapid recombination of photogenerated electron–hole pairs, severely compromising its photocatalytic efficiency [4,5,6].

Elemental doping is a well-established and effective strategy to modulate the electronic band structure of semiconductors and extend the light response range [7,8,9]. Calcium (Ca), an alkaline earth metal, has an ionic radius (Ca^2+^: 100 pm) that is well-matched to that of Zn^2+^ (74 pm). Doping with Ca can induce lattice distortion and introduce impurity energy levels without disrupting the parent crystal structure. However, conventional coprecipitation methods for the synthesis of nano-ZnO typically require a large dosage of alkaline reagents, and the resulting products are prone to severe particle agglomeration. Therefore, the development of green, low-consumption synthetic routes for ZnO nanomaterials is of great research significance. Cactus juice, which is rich in polysaccharides, organic acids, and mineral elements, can act as a natural complexing agent and precipitation medium, potentially enabling the controllable synthesis and surface modification of nanomaterials in an eco-friendly manner [10,11].

In this study, we present, for the first time, a green and efficient method for the in situ synthesis of Ca-doped ZnO nanosheets using cactus juice as an auxiliary precipitant. Among various plant extracts for green nanomaterial synthesis, cactus juice was selected as the reaction medium due to its unique advantages over common alternatives. It is rich in synergistic polysaccharides and organic acids, enabling one-step controllable synthesis of ZnO nanosheets without extra additives. Naturally containing Ca and other minerals, it serves as an intrinsic doping source for simultaneous morphology control and doping. Additionally, cactus is sustainable and low-cost, with juice easily obtained via simple squeezing and filtration, avoiding complex extraction. Free of toxic metabolites, it is more promising for large-scale photocatalytic material synthesis. We systematically investigated the effect of Ca doping concentration on the structural, morphological, optical, and photocatalytic properties of the as-prepared samples. Through comprehensive spectroscopic and photoelectrochemical analysis, we elucidated the underlying mechanism through which Ca doping and the concomitant formation of oxygen vacancies dramatically enhance the visible-light-driven hydrogen evolution performance.

## 2. Results and Discussion

### 2.1. Material Structure and Morphology

**Figure 1:**
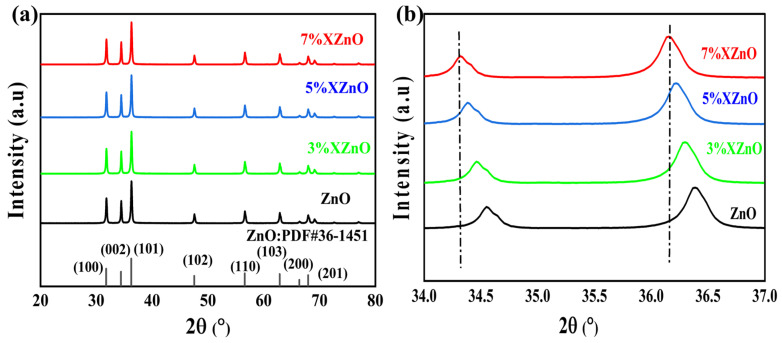
(**a**) XRD patterns of the prepared XZnO samples and (**b**) magnified view of the (002) and (101) peaks.

The crystalline structure of the synthesized samples was first investigated by X-ray diffraction (as shown in Figure 1). All diffraction peaks of both pristine and Ca-doped ZnO samples are perfectly indexed to the hexagonal wurtzite phase (PDF#36-1451), with no characteristic peaks assignable to CaO or other calcium-based compounds. This result confirms that in situ Ca doping, facilitated by the cactus juice medium, does not disrupt the intrinsic crystal lattice of ZnO. A magnified view of the (002) and (101) diffraction peaks (Figure 1b) shows that the diffraction peaks of the XZnO samples shift slightly towards lower angles compared with those of pristine ZnO. In accordance with Bragg’s law, the decrease in diffraction angles points to an increased lattice spacing. This expansion arises from the partial displacement of native Zn^2+^ (ionic radius: 74 pm) by the larger Ca^2+^ dopant (100 pm), thereby verifying the effective doping of Ca into the ZnO lattice.

**Figure 2:**
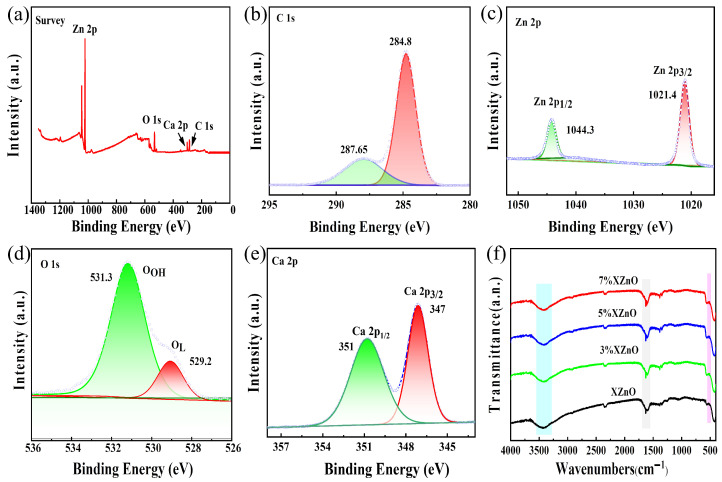
(**a**) XPS survey spectrum, (**b**) C 1s, (**c**) Zn 2p, (**d**) O 1s, (**e**) high-resolution Ca 2p XPS spectra of XZnO samples, and (**f**) FT-IR spectra of the prepared XZnO samples.

X-ray photoelectron spectroscopy (XPS) was performed to analyze the surface chemical composition and elemental valence states of the as-prepared samples. The survey spectra in Figure 2a,b confirm the presence of Zn and O in all samples, as well as Ca in the doped samples, with no impurity signals detected. The high-resolution Zn 2p spectrum (Figure 2c) exhibits two symmetric characteristic peaks at 1021.4 eV and 1044.3 eV, corresponding to Zn 2p_3/2_ and Zn 2p_1/2_, respectively, with a spin–orbit splitting of 22.9 eV, which is characteristic of the Zn^2+^ oxidation state. The high-resolution O 1s spectrum (Figure 2d) can be fitted with two components: a peak at 529.2 eV attributed to lattice oxygen (O_L_) and a peak at 531.3 eV associated with surface hydroxyl groups (O_OH_) or oxygen-deficient regions. The successful incorporation of Ca is unambiguously confirmed by the high-resolution Ca 2p spectrum (Figure 2e), which shows two well-resolved doublet peaks at approximately 347 eV and 351 eV, corresponding to Ca 2p_3/2_ and Ca 2p_1/2_, and confirming calcium’s presence as Ca^2+^.

Fourier-transform infrared (FT-IR) spectroscopy, shown in Figure 2f, further supports these findings. All samples exhibit a strong absorption band around 500 cm^−1^, characteristic of the Zn-O stretching vibration. Notably, this peak becomes sharper and more intense with increasing Ca content, which can be attributed to Ca^2+^ substitution into the ZnO lattice, leading to the enhancement of local Zn-O bond strength and overall structural rigidity of the nanosheets. The broad band around 3500 cm^−1^ corresponds to the O-H stretching vibration of adsorbed water molecules and surface hydroxyl groups. The peak at 1650 cm^−1^ is dominated by the H-O-H in-plane bending vibration of physically adsorbed water, with a minor overlapping contribution from the bending vibration of surface hydroxyl groups. The increase in intensity of the 1650 cm^−1^ peak with Ca doping is particularly significant, indicating that Ca doping effectively improves the surface hydrophilicity of the sample. A more hydrophilic surface with abundant adsorbed water and surface hydroxyl groups is known to facilitate the adsorption and activation of water molecules, a crucial initial step in the photocatalytic water-splitting reaction.

**Figure 3:**
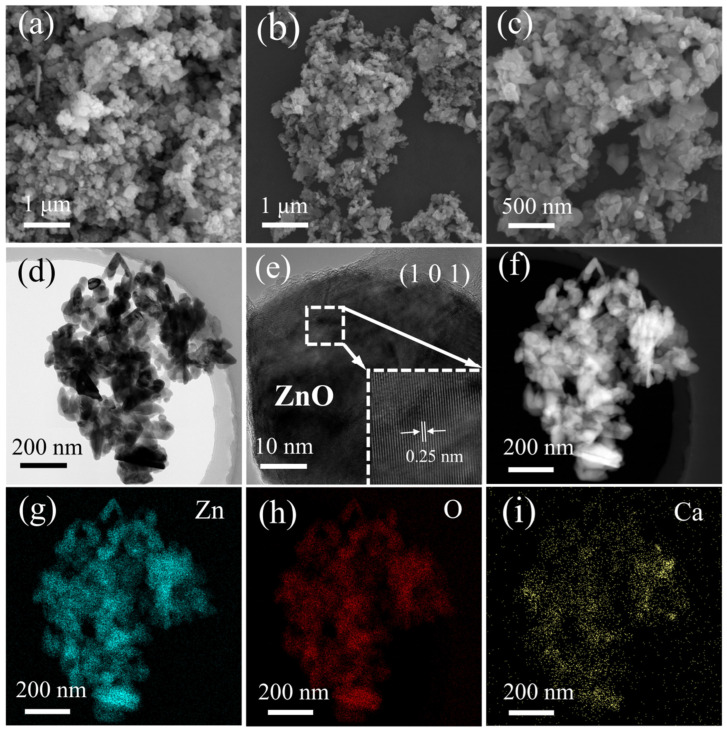
(**a**–**c**) TEM images, (**d**–**f**) HRTEM images, and (**g**–**i**) EDS elemental mapping of the 5%XZnO sample.

The morphology and microstructure of the optimal 5%XZnO sample were examined using transmission electron microscopy (TEM). The TEM images in Figure 3a–c reveal a well-defined, uniformly dispersed nanosheet morphology with negligible agglomeration, highlighting the effectiveness of the cactus-juice-mediated synthesis in controlling particle growth. High-resolution TEM (HRTEM) in Figure 3d,e displays clear and continuous lattice fringes with an interplanar spacing of 0.25 nm, which is well consistent with the (101) crystal plane of wurtzite ZnO. Energy-dispersive X-ray spectroscopy (EDS) elemental mapping (Figure 3g–i) reveals the homogeneous distribution of Zn, O, and Ca across the entire nanosheet. No Ca-rich clusters or secondary phases are observed, which provides solid evidence that Ca^2+^ ions are uniformly incorporated into the ZnO lattice, rather than merely adsorbed on the sample surface or forming isolated CaO domains. Furthermore, the calcium content in the as-prepared XZnO sample was quantitatively determined via energy-dispersive X-ray spectroscopy (EDS) elemental analysis, with the corresponding characterization results presented in Figure S1. Specifically, Figure S1a shows the high-angle annular dark-field (HAADF) image of the XZnO sample, Figure S1b displays the elemental content of each component in the sample, and Figure S1c summarizes the detailed quantitative elemental analysis data. Based on the EDS results, the mass fraction of calcium in the XZnO sample is calculated to be approximately 0.58 wt%.

### 2.2. Optical Properties and Band Structure Evolution

**Figure 4:**
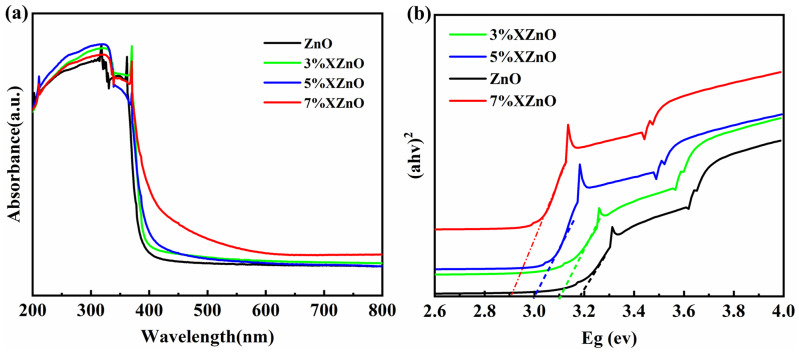
(**a**) UV-vis diffuse reflectance spectra and (**b**) corresponding Tauc plots for bandgap energy determination of the XZnO samples.

To investigate the influence of Ca incorporation on the optical characteristics of the prepared samples, UV-vis diffuse reflectance spectroscopy (DRS) was employed. As presented in Figure 4a, the spectrum of undoped ZnO exhibits a sharp absorption threshold located at around 390 nm within the ultraviolet region, while its response to visible light remains minimal. Upon Ca doping, a progressive red-shift of the absorption edge is observed, accompanied by a significant increase in visible light absorption. The corresponding bandgap energies, determined from Tauc plots (Figure 4b), decrease monotonically from 3.19 eV for pure ZnO to 2.90 eV for the 7% XZnO sample. This bandgap narrowing is attributed to the formation of impurity energy levels within the ZnO bandgap due to the substitutional Ca^2+^ ions, effectively lowering the energy required for electronic transitions and extending the light absorption range into the visible spectrum.

**Figure 5:**
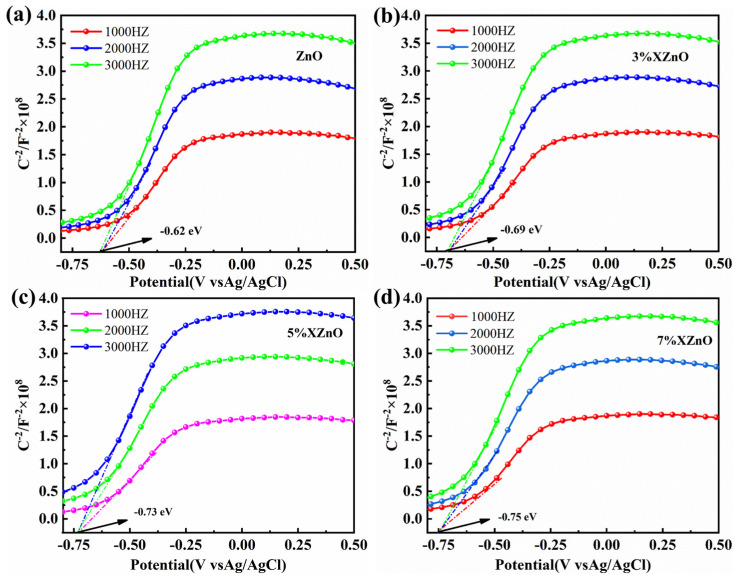
Mott–Schottky plots of (**a**) ZnO, (**b**) 3%XZnO, (**c**) 5%XZnO, and (**d**) 7%XZnO.

To elucidate the absolute band edge positions, Mott–Schottky (M-S) measurements were performed (Figure 5). The positive slopes of the linear plots confirm the n-type semiconductor nature. The flat-band potentials (E_FB_), which for n-type semiconductors are approximately equal to the conduction band minimum (E_CB_), were determined from the x-intercepts. After conversion to the standard hydrogen electrode (SHE) scale, the E_CB_ values are −0.42 V for pure ZnO, −0.49 V for 3%XZnO, −0.53 V for 5%XZnO, and −0.55 V for 7% XZnO. Using the bandgap energies (E_g_) obtained from DRS, the valence band maxima (E_VB_ = E_CB_ + E_g_) were calculated. The progressive negative shift of E_CB_ with increasing Ca content is particularly noteworthy, as it implies that the photogenerated electrons possess a stronger thermodynamic driving force for the proton reduction reaction (H^+^/H_2_), a key factor in enhancing photocatalytic hydrogen production.

### 2.3. Photocatalytic Hydrogen Production Performance

**Figure 6:**
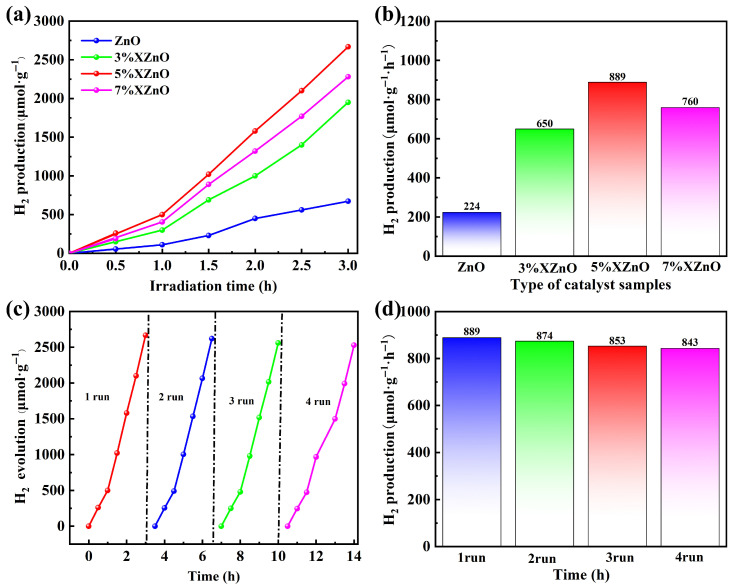
(**a**) Time-dependent photocatalytic hydrogen production and (**b**) corresponding hydrogen evolution rates of the XZnO samples. (**c**) Cycling stability test and (**d**) relative activity retention of the 5%XZnO sample.

The photocatalytic activity of the synthesized samples for hydrogen evolution was evaluated under full-spectrum irradiation. As shown in Figure 6a,b, pure ZnO exhibits negligible H_2_ production, consistent with its wide bandgap and inability to absorb visible light. In stark contrast, all Ca-doped XZnO samples demonstrate significant and sustained H_2_ evolution. The hydrogen evolution rate follows a volcano-type trend with doping concentration, initially increasing, reaching a maximum, and then decreasing. The 5%XZnO sample achieves the highest performance, with an impressive H_2_ evolution rate of 889 μmol·g^−1^·h^−1^, which is about 4 times that of the pristine ZnO. The hydrogen production performance of this work is compared with that of other similar published studies in Table S1 [12,13,14,15,16,17]. This optimal doping level represents a balance between enhanced light absorption and charge separation and the introduction of excessive defects that can act as recombination centers, as observed for the 7%XZnO sample.

The stability of a photocatalyst is paramount for practical applications. The 5%XZnO sample was subjected to four consecutive cycling runs, totaling 14 h of illumination (Figure 6c). The catalyst exhibited evident stability, retaining 94.8% of its initial H_2_ evolution rate in the fourth cycle (Figure 6d). Post-reaction analyses verified that the crystal structure and surface functional groups of the catalyst remained intact, revealing its favorable stability against photocorrosion and structural degradation. Such an advantage arises from the robust lattice structure induced by Ca doping.

### 2.4. Photoelectrochemical Properties and Mechanism for Enhanced Activity

**Figure 7:**
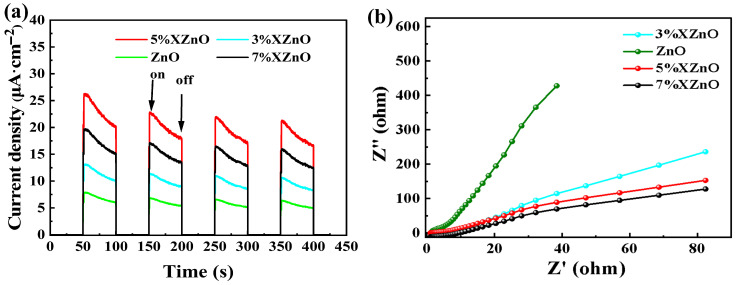
(**a**) Transient photocurrent responses and (**b**) electrochemical impedance spectroscopy (EIS) Nyquist plots of the XZnO samples.

To gain deeper insight into the charge carrier dynamics, transient photocurrent responses and electrochemical impedance spectroscopy (EIS) were performed. Figure 7a exhibits the photocurrent responses under intermittent full-spectrum irradiation. The 5%XZnO sample generates the highest photocurrent density, which is more than five times that of pure ZnO. This result confirms that appropriate Ca doping significantly promotes the separation and migration of photogenerated charge carriers. The EIS Nyquist plots shown in Figure 7b provide further support for this conclusion. The 5%XZnO sample exhibits the smallest arc radius among all samples, indicating its higher conductivity and lower charge-transfer resistance across the electrode interface. These features favor faster interfacial charge migration kinetics, enabling more photogenerated electrons to participate in the surface reduction reaction for hydrogen evolution [18,19,20].

**Figure 8:**
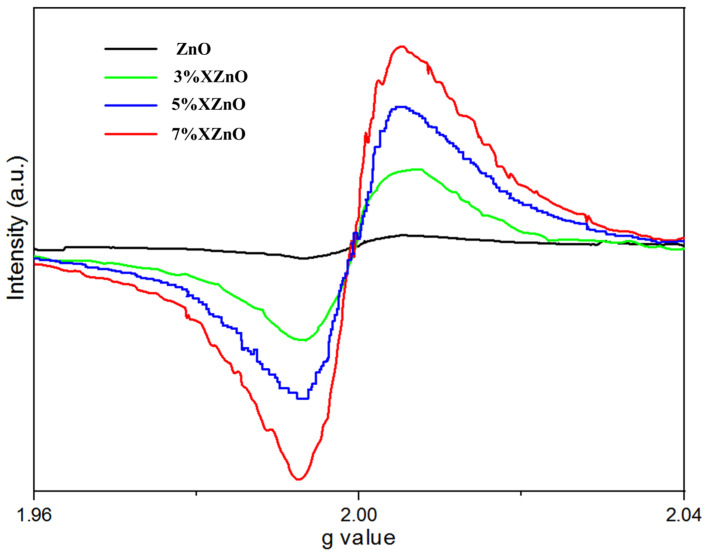
EPR spectra of pure ZnO and Ca-doped XZnO samples, revealing the variation in oxygen vacancy concentration.

To directly identify and semi-quantify oxygen vacancies, electron paramagnetic resonance (EPR) spectroscopy was employed at 77 K, with the results shown in Figure 8. A distinct signal at g ≈ 2.0, characteristic of singly ionized oxygen vacancies (O_v_), is observed for all samples. Crucially, the intensity of this signal increases with Ca doping, being weakest for pure ZnO and strongest for the 7%XZnO sample, before slightly decreasing for the 5%XZnO sample. This trend indicates that the substitution of Zn^2+^ by Ca^2+^ introduces a degree of charge imbalance and lattice strain, which facilitates the formation of oxygen vacancies. The higher concentration of oxygen vacancies in the optimally doped sample can act as shallow electron donors and efficient trap sites, temporarily immobilizing photogenerated electrons and thereby prolonging their lifetime by suppressing rapid recombination with holes. The subsequent slight decrease in the 7% sample may be due to an overabundance of defects that begin to aggregate or form different types of recombination centers. These findings provide direct evidence that the enhanced performance of the Ca-doped samples is linked not only to bandgap narrowing but also to the strategic introduction of beneficial oxygen vacancies.

**Figure 9:**
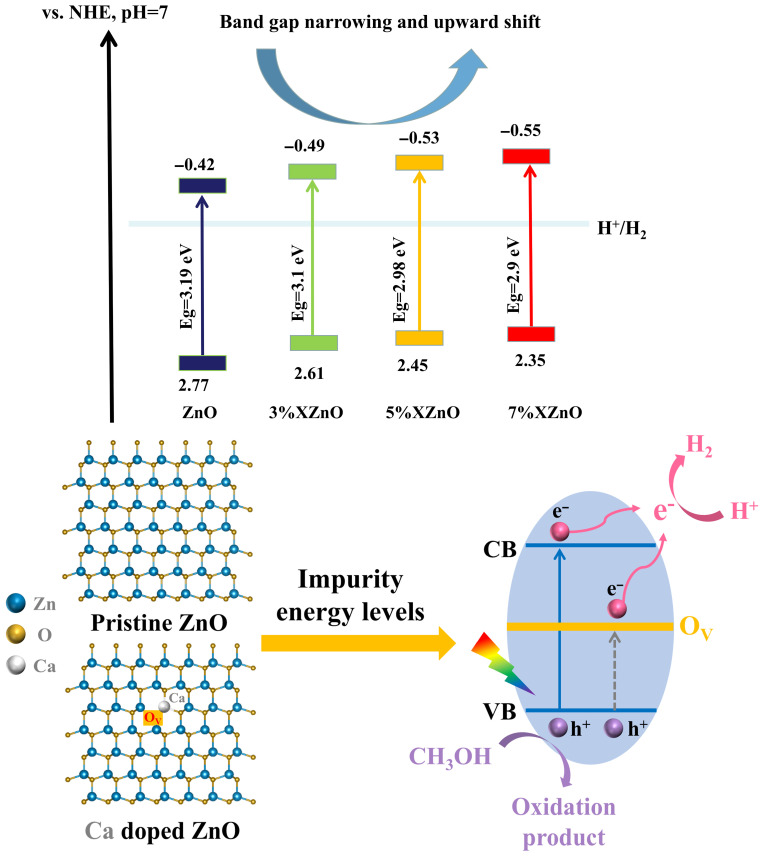
Proposed mechanism for enhanced photocatalytic hydrogen production using in situ Ca-doped ZnO nanosheets.

Based on the comprehensive experimental evidence, a plausible mechanism for the enhanced photocatalytic activity of Ca-doped ZnO nanosheets is proposed, as illustrated in Figure 9. The enhanced performance is a result of several synergistic effects: (i) Extended light absorption: Substitutional Ca^2+^ doping narrows the bandgap, enabling the absorption of full-spectrum irradiation and generating more electron–hole pairs. (ii) Enhanced charge separation: The incorporation of Ca^2+^ induces lattice distortion and promotes the formation of oxygen vacancies. These vacancies can serve as efficient electron traps, temporarily immobilizing electrons and significantly prolonging their lifetime by impeding rapid recombination with holes in the valence band. (iii) Favorable band edge positions: Ca doping shifts the conduction band minimum to more negative potentials, increasing the reducing power of the photogenerated electrons. (iv) Improved surface reactivity: The increased surface hydroxyl groups, as evidenced by FT-IR, enhance the catalyst’s hydrophilicity and its ability to adsorb water molecules, the primary reactant. These combined factors funnel a greater number of long-lived, highly energetic electrons to the surface-active sites to efficiently reduce adsorbed protons to molecular hydrogen. Furthermore, optimal Ca doping suppresses the formation of lattice defects that can act as recombination centers, improving the lifetime and migration efficiency of photogenerated charge carriers. These synergistic effects collectively contribute to the efficient and stable photocatalytic water splitting for hydrogen production by in situ Ca-doped ZnO under full-spectrum irradiation.

## 3. Experimental Section

### 3.1. Material Preparation

Briefly, 20 g of zinc acetate dihydrate (Zn(CH_3_COO)_2_·2H_2_O, ≥99%, Shanghai Aladdin Biochemical Technology Co., Ltd., Shanghai, China) was dissolved in distilled water. Separately, a 5 M NaOH (≥98%, Shanghai Aladdin Biochemical Technology Co., Ltd.) solution was prepared. According to the stoichiometric ratio, 43 mL of the NaOH solution was added dropwise to the zinc acetate solution, forming a Zn(OH)_2_ precipitate. The product was washed multiple times with distilled water, dried at 80 °C for 24 h, ground, and then calcined in a muffle furnace at 420 °C for 3 h. The resulting powder was washed, filtered, and dried again to obtain 7.10 g of white pristine ZnO (hereafter referred to as ZnO for brevity). The corresponding yield was 95.8%.

**Figure 10:**
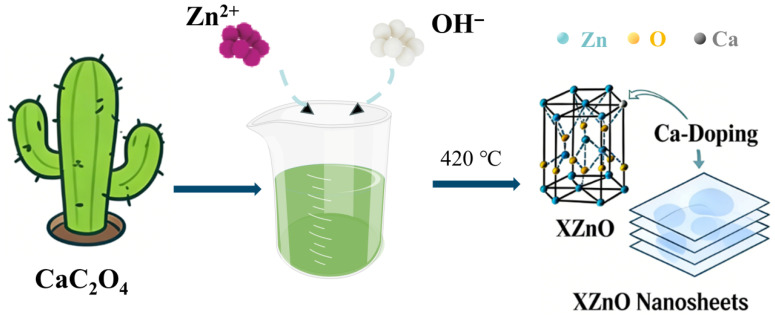
Schematic illustration of the preparation process for Ca-doped ZnO (XZnO).

An eco-friendly coprecipitation method was employed (as illustrated in Figure 10). 20 g of zinc acetate was dissolved in 200 mL of distilled water. Separately, 40 g of NaOH was dissolved in 200 mL of distilled water (5 M). Then, 19 mL of the NaOH solution (less than half of the conventional dosage) was added dropwise to the zinc acetate solution, simultaneously with 200 mL of cactus juice (The main component is CaC_2_O_4_, extracted from self-squeezed juice of Opuntia milpa alta from Mexico), which served as the precipitation medium. The mixture was stirred for 24 h. The resulting precipitate was washed, filtered, dried at 80 °C for 24 h, ground, and calcined at 420 °C for 3 h. After further washing, filtration, and drying, 9.30 g of XZnO powder was obtained. The corresponding yield of the reaction reached 98.2%. By adjusting the amount of cactus juice added, the in situ Ca doping ratio was controlled and set at 3%, 5%, and 7% (denoted as 3%XZnO, 5%XZnO, and 7%XZnO, respectively).

### 3.2. Sample Characterization

The characterization of materials is detailed in the Supporting Information.

### 3.3. Photocatalytic Hydrogen Production Performance Test

The photocatalytic performance tests of the catalyst are detailed in the Supporting Information.

### 3.4. Photoelectrochemical Measurements

Photoelectrochemical measurements, including photocurrent response, electrochemical impedance spectroscopy (EIS), and Mott–Schottky (M-S) plots, were performed using an electrochemical workstation with a standard three-electrode configuration. The working electrode was prepared by coating the catalyst onto FTO conductive glass. The electrolyte was a 0.5 M Na_2_SO_4_ solution. The light source was a 300 W Xe lamp. EIS was measured over a frequency range of 0.1 Hz to 100 kHz. M-S plots were collected at frequencies of 1000 Hz, 2000 Hz, and 3000 Hz.

## 4. Conclusions

In conclusion, a series of Ca-doped ZnO nanosheet photocatalysts was successfully fabricated via a green, eco-friendly coprecipitation strategy using cactus juice as the natural multifunctional precipitation medium. This synthetic route features the merits of reduced alkali consumption, mild conditions, and environmental friendliness. Ca^2+^ ions were successfully incorporated into the ZnO lattice. The doped samples retained the wurtzite structure, exhibited a uniformly dispersed nanosheet morphology with clear lattice fringes, and showed a homogeneous distribution of Ca without any separate impurity phases. Ca doping effectively narrowed the bandgap of ZnO (from 3.19 eV to 2.90 eV), extended its full-spectrum irradiation absorption range, and induced a negative shift of the conduction band minimum, thus enhancing the reduction ability of photogenerated electrons. Meanwhile, Ca doping also promoted the formation of oxygen vacancies, which served as electron traps to significantly boost the separation of photogenerated charge carriers and suppress recombination. The 5%XZnO sample demonstrated the optimal visible-light-driven photocatalytic hydrogen production performance, with a rate of 889 μmol·g^−1^·h^−1^ and excellent cyclic stability (maintaining 94.8% activity after four cycles). Photoelectrochemical measurements confirmed that an appropriate amount of Ca doping significantly promoted charge carrier separation and interfacial migration. This work provides a novel avenue for the green synthesis of high-efficiency and stable ZnO-based visible-light photocatalysts, and the proposed eco-friendly coprecipitation method possesses good universality and promising potential for practical application.

## Data Availability

The original contributions presented in this study are included in the article/Supplementary Material. Further inquiries can be directed to the corresponding author(s).

## Supplementary Materials

The following supporting information can be downloaded at: https://www.mdpi.com/article/10.3390/molecules31071091/s1, Figure S1. (a) HAADF-STEM image of XZnO, (b) Figure X EDS spectrum of the XZnO sample, (c) Corresponding EDS spectrum data; Table S1. Comparison of H_2_ production among different photocatalysts.
