# Supplementary material for: Green Synthesis of Ca-Doped ZnO Nanosheets with Tunable Band Structure via Cactus-Juice-Mediated Coprecipitation for Enhanced Photocatalytic H2 Evolution
Source: Molecules. 2026 Mar 26;31(7):1091. doi: 10.3390/molecules31071091 (PMC13074849; doi:10.3390/molecules31071091)
Supplement: Supplementary file 1 [file molecules-31-01091-s001.zip › molecules-4213928-supplementary.pdf]

Supporting Information for

Green Synthesis of Ca-Doped ZnO Nanosheets with Tunable Band Structure via Cactus-Juice-Mediated Coprecipitation for Enhanced Photocatalytic H_2 Evolution

Heji Luo[1, #], Huifang Liu[1, #], Simin Liu[1], Haiyan Wang[1], Lingling Liu[2], Xibao Li[1, 2, *]

[1]School of Materials Science and Engineering, Nanchang Hangkong University, Nanchang 330063, China

[2]College of Environment and Chemical Engineering, Nanchang Hangkong University, Nanchang 330063, China

[#]These authors contributed equally to this work

[*]Corresponding authors. E-mail: lixibao@nchu.edu.cn.

1. Experimental section

1.1 Sample Characterization

The crystal structure of the samples was analyzed using X-ray diffraction (XRD, Cu Kα radiation, $\lambda = 0.15406$ nm). The surface chemical states were analyzed by X-ray photoelectron spectroscopy (XPS, Al Kα source), with binding energies calibrated using the C 1s peak (284.8 eV). Sample morphology, particle size, and lattice fringes were observed using transmission electron microscopy (TEM) and high-resolution transmission electron microscopy (HRTEM). Energy-dispersive X-ray spectroscopy (EDS) was used for elemental distribution mapping. Fourier-transform infrared spectroscopy (FT-IR) was employed to analyze surface functional groups. UV-vis diffuse reflectance spectroscopy (UV-vis DRS) was used to measure light absorption properties, and the bandgap energy was calculated using the Tauc plot method.

1.2 Photocatalytic Hydrogen Production Performance Test

Photocatalytic hydrogen evolution experiments were conducted in a closed-circulation system. 50 mg of the catalyst was dispersed in 100 mL of an aqueous solution containing 10 vol% CH_3OH (as a sacrificial agent). A 300 W Xe lamp was used as the full-spectrum light source. Prior to the reaction, the system was purged with nitrogen for 30 min to remove dissolved oxygen. The evolved hydrogen gas was quantified using a gas chromatograph equipped with a thermal conductivity detector (TCD) and high-purity argon carrier gas. Cyclic stability tests were performed for four consecutive runs, each lasting 4 h.

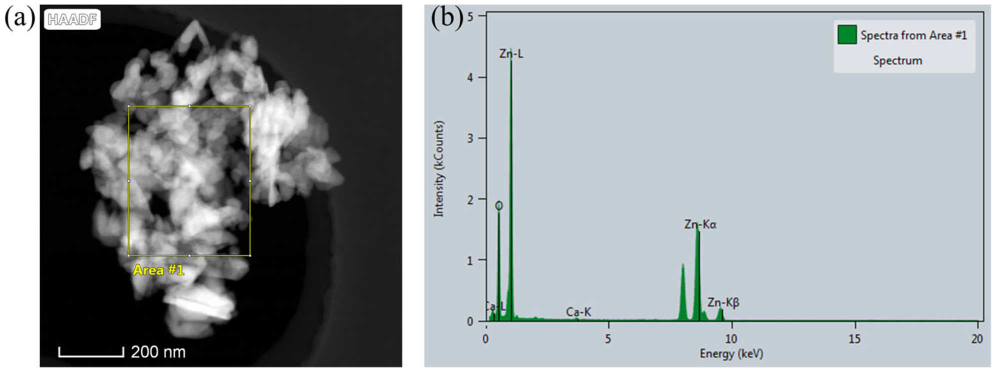

(c)

Element	Atomic Fraction(%)	Atomic Error(%)	Mass Fraction(%)	Mass Error(%)	Fit error (%)
O	58.60	8.77	25.81	2.55	0.80
Ca	0.52	0.11	0.58	0.10	1.68
Zn	40.88	8.19	73.61	12.20	0.42

Figure S1. (a) HAADF-STEM image of XZnO, (b) Figure X EDS spectrum of the XZnO sample, (c) Corresponding EDS spectrum data.

Table S1 Comparison of H_2 production among different photocatalysts

Material	Sacrificial reagent	H_2 evolution rate ($\mu mol\cdot g^{-1}\cdot h^{-1}$)	Ref.
**5%XZnO**	**CH_3OH**	**889**	**This work**
Cu-ZnO	Glycerol	433	[1]
Ca-ZnO	CH_3OH	234	[2]
ZnO/red phosphorus	TEOA	99	[3]
Ag-ZnO	Na_2S and Na_2SO_3	712	[4]
Co_3O_4/ZnO	Glycerol	513	[5]
Nitrogen-rich ZnO/g-C_3N_4	TEOA	780	[6]

References

[1] Vaiano, V.; Iervolino, G. Photocatalytic hydrogen production from glycerol aqueous solution using Cu-doped ZnO under visible light irradiation. Appl. Sci. **2019**, 9, 2741.

[2] Ahmad, I.; Ahmed, E.; Ahmad, M.; Muhammad, S.; Muhammad A.; Waheed Q.; Muahmmad I.; Absar A.; Mian F. The investigation of hydrogen evolution using Ca doped ZnO catalysts under visible light illumination. Mat. Sci. Semicon. Proc. **2020**, 105, 104748.

[3] Chen, J.; Huang, S.; Long, Y.; Wu, J.; Li, H.; Li, Z.; Zeng, Y.; Ruan, S. Fabrication of ZnO/red phosphorus heterostructure for effective photocatalytic H_2 evolution from water splitting. Nanomaterials **2018**, 8, 835.

[4] Nethravathi, P.; Suresh, D. Silver-doped ZnO embedded reduced graphene oxide hybrid nanostructured composites for superior photocatalytic hydrogen generation, dye degradation, nitrite sensing and antioxidant activities. Inorg. Chem. Commun. **2021**, 134, 109051.

[5] Mohamed, R.; Shawky, A. Visible-light-driven hydrogen production over ZIF-8 derived Co_3O_4/ZnO S-scheme based p-n heterojunctions. Opt. Mater. **2022**, 124, 112012.

[6] Liu, X.; Liu, L.; Yao, Z.; Yang, Z.; Xu, H. Enhanced visible-light-driven photocatalytic hydrogen evolution and NO photo-oxidation capacity of ZnO/g-C_3N_4 with N dopant. Colloids and Surfaces A: Physicochemical and Engineering Aspects, **2020**, 599, 124869.
